# Retraction Note: Investigation of molecular biomarker candidates for diagnosis and prognosis of chronic periodontitis by bioinformatics analysis of pooled microarray gene expression datasets in Gene Expression Omnibus (GEO)

**DOI:** 10.1186/s12903-024-04383-7

**Published:** 2024-05-27

**Authors:** Asami Suzuki, Tetsuro Horie, Yukihiro Numabe

**Affiliations:** 1 General Dentistry, The Nippon Dental University Hospital at Tokyo, 2-3-16 Fujimi, Chiyoda-ku, Tokyo, 102-8158 Japan; 2 Research Center for Odontology, The Nippon Dental University at Tokyo, 1-9-20 Fujimi, Chiyoda-ku, Tokyo, 102-0071 Japan; 3 Department of Periodontology, The Nippon Dental University at Tokyo, 1-9-20 Fujimi, Chiyoda-ku, Tokyo, 102-0071 Japan

**Retraction note: BMC Oral Health (2019) 19:52**

10.1186/s12903-019-0738-0

The Editor has retracted this article after concerns were raised about the data reported. Two of the datasets used in this analysis, GSE10334 and GSE16134, were generated from the same archive of healthy and diseased gingival tissues, and as such there is a significant sampling overlap between the two. The authors do not appear to have been aware of this limitation when they pooled these datasets for this study. The Editor no longer has confidence in the reliability of the article’s results and conclusions.

Asami Suzuki has stated on behalf of all authors that they agree with this retraction.

